# Ligand Screening and Discovery Using Cocktail Soaking and Automated Microcrystal Electron Diffraction

**DOI:** 10.1002/cmdc.202500156

**Published:** 2025-04-11

**Authors:** Jieye Lin, Marc J. Gallenito, Johan Hattne, Tamir Gonen

**Affiliations:** ^1^ Department of Biological Chemistry University of California, Los Angeles 615 Charles E. Young Drive South Los Angeles CA 90095 USA; ^2^ Howard Hughes Medical Institute University of California, Los Angeles Los Angeles CA 90095 USA; ^3^ Department of Physiology University of California, Los Angeles 615 Charles E. Young Drive South Los Angeles CA 90095 USA

**Keywords:** cocktail soaking, high‐throughput data collection, ligand screening, microcrystal electron diffraction, microscale thermophoresis

## Abstract

Cocktail soaking using single‐crystal X‐ray diffraction previously allowed high‐throughput crystallographic screening of ligands against protein targets. However, protein microcrystals are not amenable to this approach if they are too small to yield strong diffraction patterns. Herein, a workflow integrating cocktail soaking with automated microcrystal electron diffraction to allow rapid ligand screening, structure determination, and binding analysis directly from microcrystals is developed. This can improve the successful hit rate, because binding is often more efficient when smaller crystals are soaked in the ligand. The approach is validated with known ligands of thermolysin and identified novel binding interactions for ligands of proteinase K. The structures of multiple protein–ligand complexes, including ligands with weak binding affinities, can be solved rapidly. Their estimated relative binding affinities are in good agreement with previous work and independent microscale thermophoresis measurements.

## Graphical abstract

Cocktail soaking using single‐crystal X‐ray diffraction allows high‐throughput crystallographic ligand screening against protein targets. However, protein microcrystals are not amenable to this approach. Herein, a workflow integrating cocktail soaking with automated microcrystal electron diffraction to allow rapid ligand screening, structure determination, and binding analysis directly from microcrystals is demonstrated.

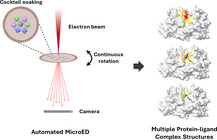

## 1 Introduction

Macromolecular crystallography remains the leading technique for 3D structural determination of protein–ligand complexes at atomic resolution, because the ligand and its interactions with the protein can be resolved from density maps.^[^ ^1^ ^]^ Crystallographic ligand screening was traditionally limited by low throughput until 1997, when Verlinde *et al.* introduced the concept of “cocktail soaking”, which significantly improved the ligand screening rates. After soaking triose‐phosphate isomerase (a glycolytic enzyme) in four cocktails of 128 antitrypanosomiasis drugs, they ultimately identified one binding hit from F_o_–F_c_ maps.^[^ ^2^ ^]^ The method has since been widely applied in drug discovery targeting various disease‐related proteins like urokinase (a kinase highly expressed in invasive cancers)^[^ ^3^ ^]^ and nucleoside 2‐deoxyribosyltransferase (related to Chaga's disease).^[^ ^4^ ^]^ Additionally, cocktail soaking can reveal allosteric binding sites and unknown functions on target proteins.^[^ ^5^, ^6^ ^]^ The efficiency of cocktail screening has been significantly enhanced with advancements in automated soaking^[^ ^7^, ^8^ ^]^ and single‐crystal X‐ray diffraction (SC‐XRD) data collection.^[^ ^8^, ^9^, ^10^ ^]^

Achieving high occupancy of ligand in crystals typically requires highly concentrated solution and long incubation time.^[^ ^11^ ^]^ The process also risks cracking or dissolving the protein crystals, which compromises the diffraction quality.^[^ ^11^ ^]^ On the contrary, soaking ligands into microcrystals is generally more effective, as their smaller crystal volumes facilitate ligand diffusion.^[^ ^12^, ^13^ ^]^ However, most microcrystals are unsuitable for conventional synchrotron‐based X‐ray diffraction due to their small sizes (<5 μm),^[^ ^14^ ^]^ and access to X‐ray free‐electron laser facilities remains globally limited.^[^ ^15^ ^]^ These shortcomings can efficiently be addressed with microcrystal electron diffraction (MicroED).

**Figure 1:**
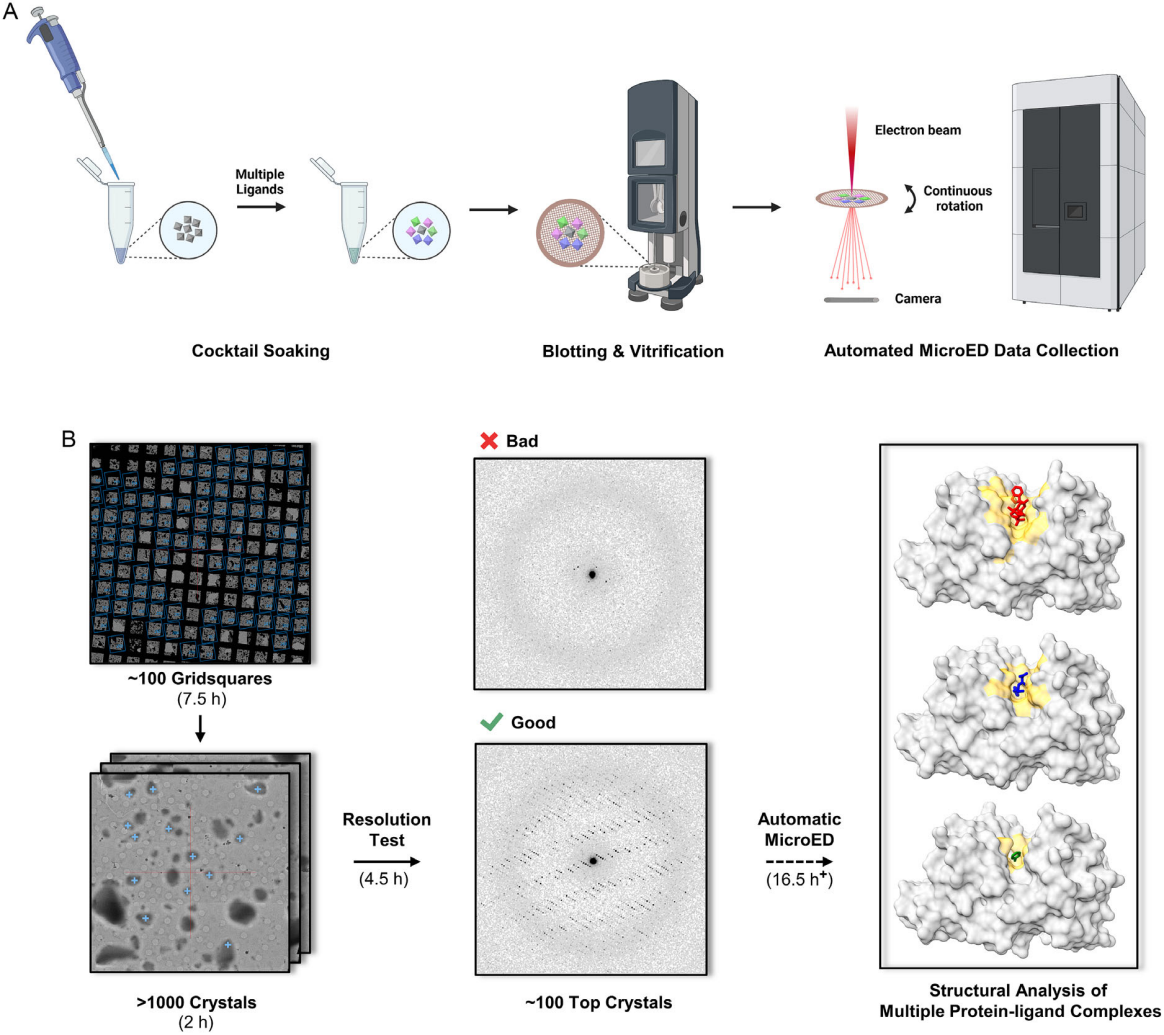
The workflow of A) cocktail soaking and B) automated MicroED data collection. Typical data collection was performed in two major steps: first, a preresolution test of over 1000 microcrystals to estimate data quality; second, automated MicroED data collection of the top‐ranked crystals.

MicroED is well‐suited for 3D structural analysis, requiring crystals only a billionth size of those needed for conventional X‐ray diffraction.^[^ ^16^, ^17^, ^18^ ^]^ MicroED has been widely adopted for elucidating the structures of pharmaceutical small molecules,^[^ ^19^, ^20^ ^]^ proteins,^[^ ^21^, ^22^ ^]^ and protein–ligand complexes.^[^ ^23^ ^]^ Automated MicroED has significantly increased throughput, such that hundreds or thousands of datasets are sequentially collected grain‐by‐grain, enabling efficient structural and compositional analysis of mixtures^[^ ^24^, ^25^ ^]^ (**Figure** **1**). MicroED is a promising method for cocktail screening, as indicated in our prior study, where the binding of 5‐amino‐2,4,6‐triodoisophthalic acid soaked into proteinase K was determined with superior efficiency and occupancy compared to larger crystals under the same condition.

**Figure 2:**
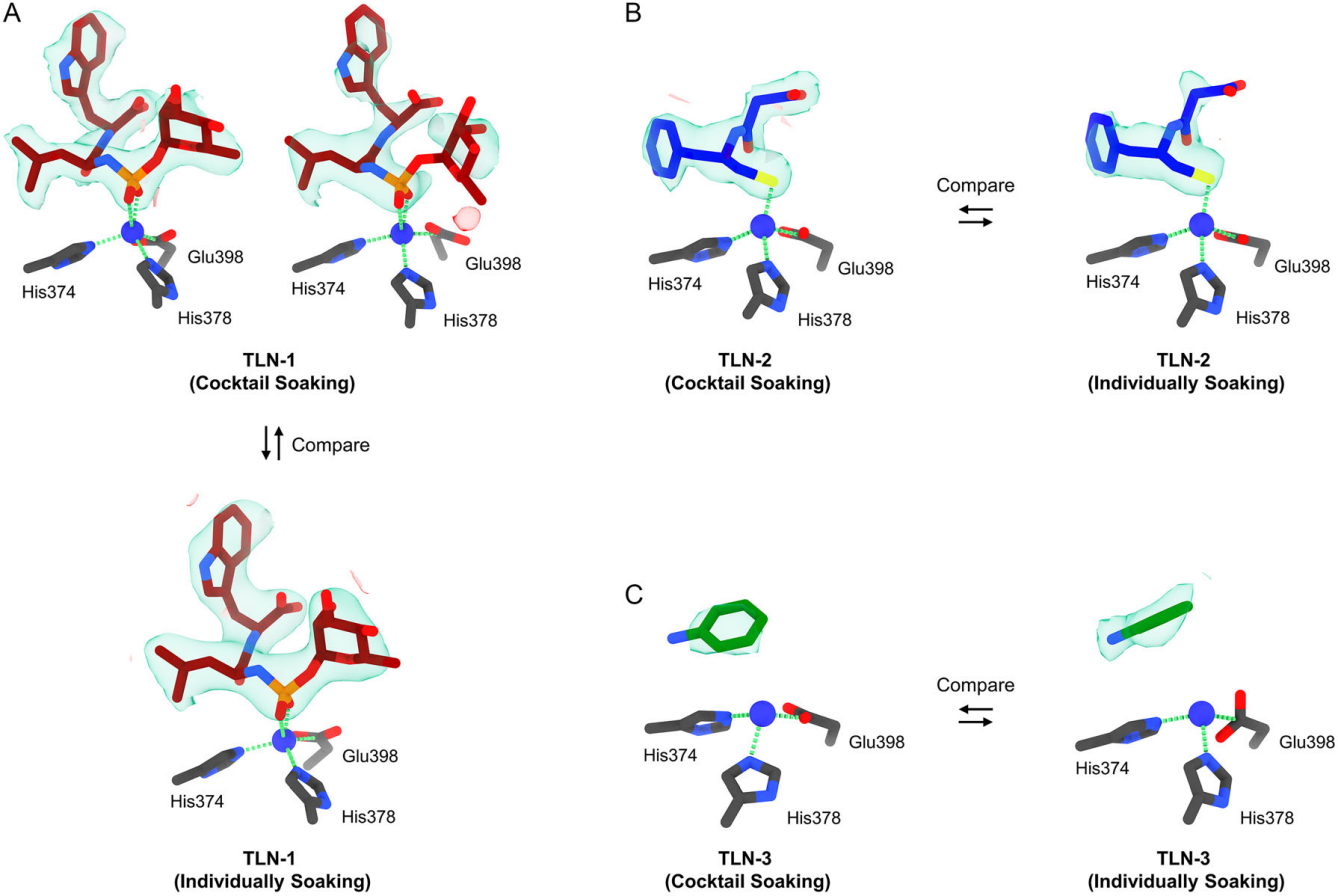
Comparison of the structure and electrostatic potential maps of **TLN**‐**1** to **TLN**‐**3** under two soaking approaches. A) Top‐left, **TLN**‐**1** from cocktail soaking set A1 (F_o_–F_c_: 2.5σ); top‐right, **TLN**‐**1** from cocktail soaking set A2 (F_o_–F_c_: 2.1σ); bottom, **TLN**‐**1** from individual soaking set B (F_o_–F_c_: 2.5σ). B) Left, **TLN**‐**2** from cocktail soaking set A2 (F_o_–F_c_: 2.5σ); right, **TLN**‐**2** from individual soaking set C (F_o_–F_c_: 2.5σ). C) Left, **TLN**‐**3** from cocktail soaking set A2 (F_o_–F_c_: 2.5σ); right, **TLN**‐**3** from individual soaking set D (F_o_–F_c_: 3.0σ). The carbon atoms for ligands **1**–**3** were colored in red, blue, and green, respectively, their F_o_–F_c_ maps were colored in green and red.

**Figure 3:**
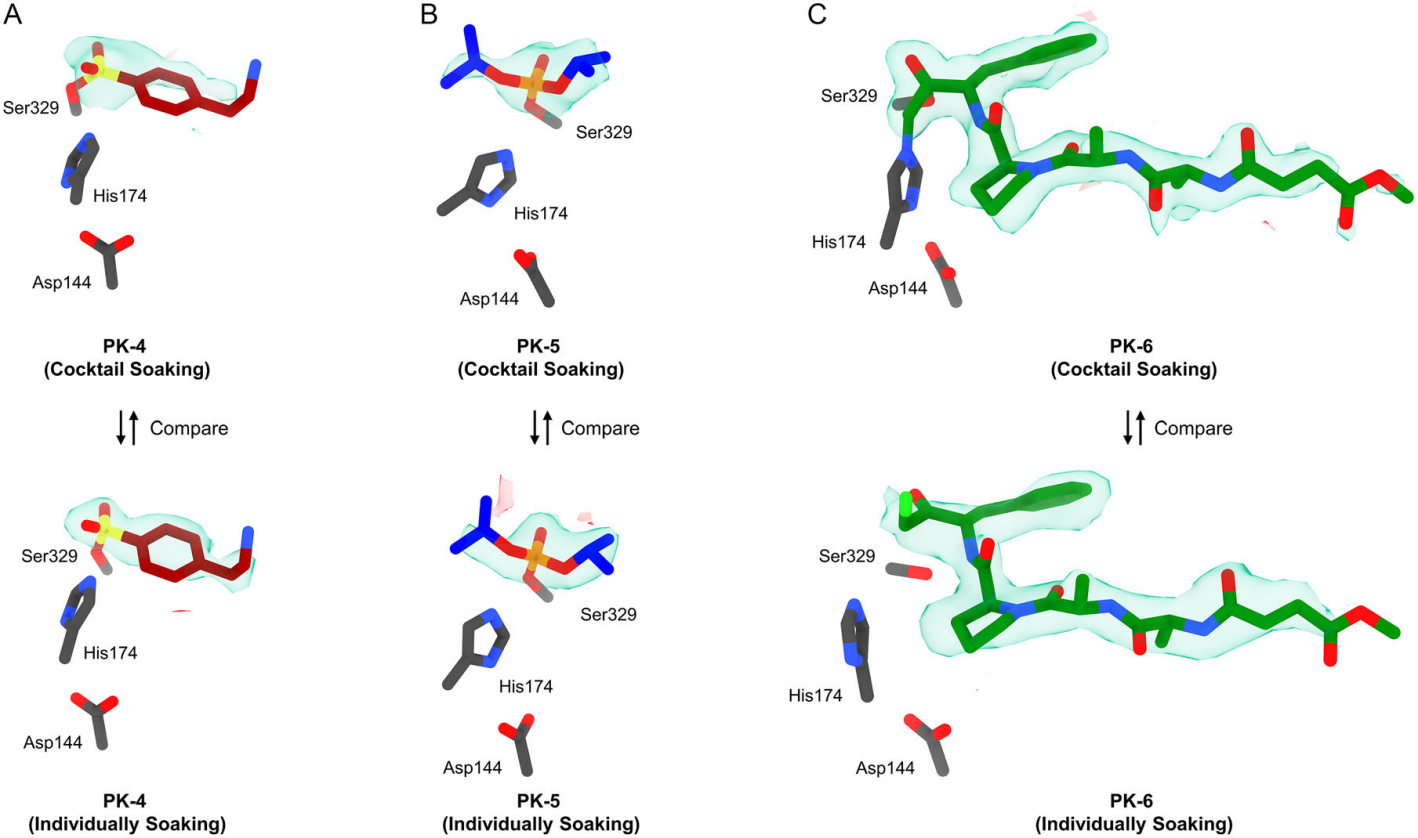
Comparison of the structure and electrostatic potential maps of **PK**‐**4** to **PK**‐**6** under two soaking approaches. A) Top, **PK**‐**4** from cocktail soaking set E (F_o_–F_c_: 2.3σ); bottom, **PK**‐**4** from individual soaking set F (F_o_–F_c_: 2.3σ). B) Top, **PK**‐**5** from cocktail soaking set E (F_o_–F_c_: 2.5σ); bottom, **PK**‐**5** from individual soaking set G (F_o_–F_c_: 2.5σ). C) Top, **PK**‐**6** from cocktail soaking set E (F_o_–F_c_: 2.5σ); bottom, **PK**‐**6** from individual soaking set H (F_o_–F_c_: 2.5σ). The carbon atoms for ligands **4**–**6** were colored in red, blue, and green, respectively, their F_o_–F_c_ maps were colored in green and red.

**Figure 4:**
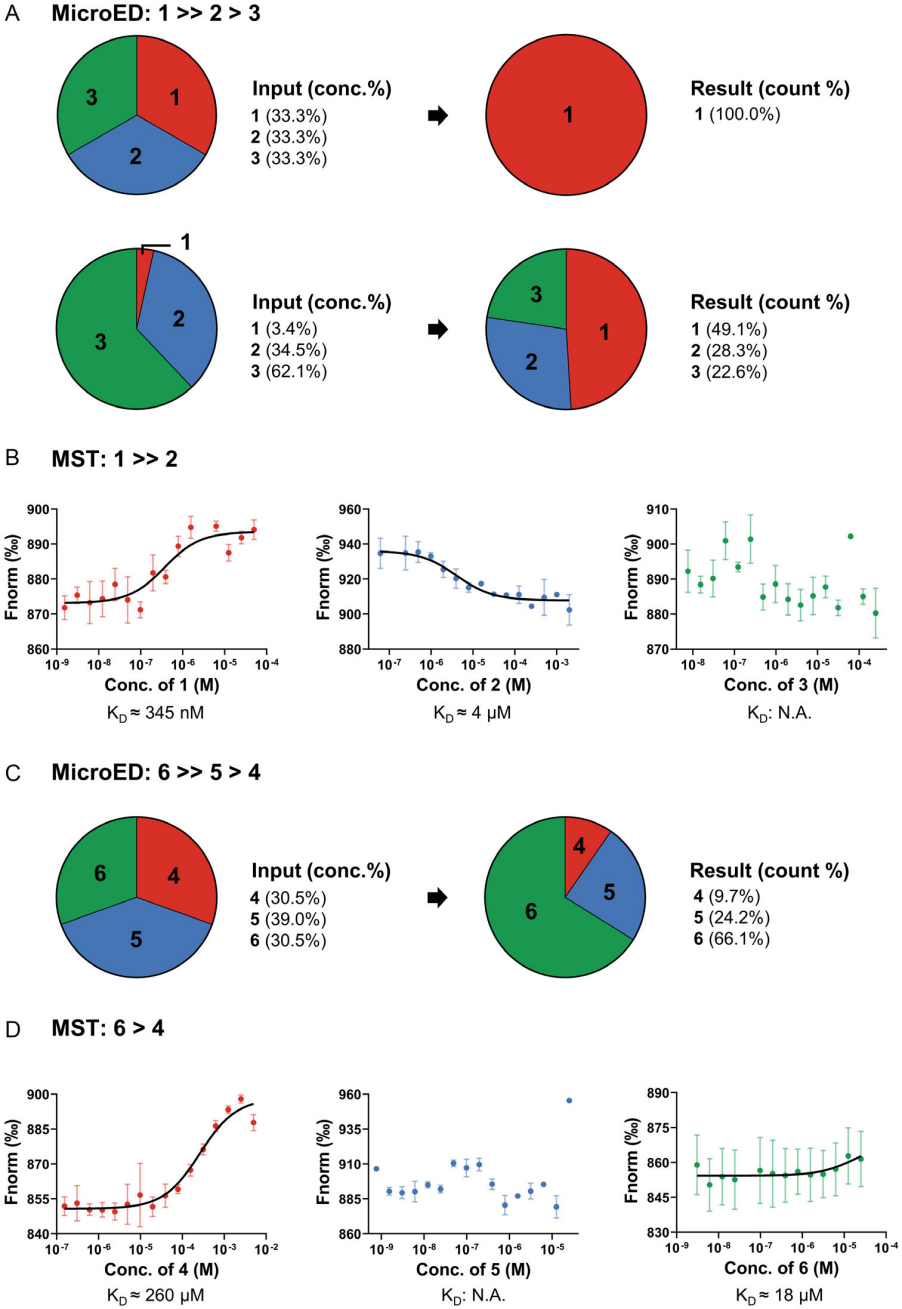
Relative binding affinities of ligands **1**–**6** determined by MicroED and MST. A) Relative binding affinities of ligands **1**–**3** determined by MicroED. B) *K*_D_ values of ligands **1**–**3** measured by MST. C) Relative binding affinities of ligands **4**–**6** determined by MicroED. D) *K*_D_ values of ligands **4**–**6** measured by MST. *K*_D_ was calculated by Equation (1) (replicate 2 times).

In this study, we used automated MicroED to rapidly screen for ligand binding in protein microcrystals soaked in ligand cocktails (Figure 1).^[^ ^24^, ^25^ ^]^ Multiple complexes were determined for the same protein and the relative binding affinities of each ligand were assessed. We first validated the workflow using three known ligands of thermolysin (**TLN**): phosphoramidon disodium salt (ligand **1**), thiorphan (ligand **2**), and aniline (ligand **3**).^[^ ^26^, ^27^, ^28^ ^]^ We then extended this workflow to uncover the unknown protein–ligand bindings of three potent ligands for proteinase K (**PK**): benzenesulfonyl fluoride hydrochloride (ligand **4**), diisopropyl fluorophosphate (ligand **5**) and a tetrapeptidyl chloromethyl ketone compound, MeOSuc‐Ala‐Ala‐Pro‐Phe‐CH_2_Cl (ligand **6**). Their resulting structures are consistent with previous studies (**Figure** **2** and **3**)^[^ ^29^, ^30^ ^]^ and our estimated relative binding affinities agree with independent microscale thermophoresis (MST) measurements (**Figure** **4**). This study demonstrates that cocktail soaking and MicroED can provide a robust means for screening ligands against protein targets.

## 2 Results

### 2.1 Method Validation: Thermolysin in Complex with Ligands **1**–**3**

Thermolysin (**TLN)** is a protein originally isolated from *Bacillus thermoproteolyticus*,^[^ ^31^ ^]^ containing a Zn ion coordinated by His374, Glu398, and His478 residues in its active sites (Figure S2, Supporting Information).^[^ ^32^, ^33^ ^]^ **TLN** can form complexes with three known ligands **1**–**3** (PDB entries: 1TLP, 1Z9G, 3MS3).^[^ ^26^, ^27^, ^28^ ^]^ Soaking **TLN** in a cocktail of ligands **1**–**3** is expected to yield crystals in which the active sites are occupied according to the binding affinities (*K*_D_) and concentrations of the respective ligands, since all the ligands bind noncovalently to the same site (Figure S1A and S2A, Supporting Information).

In set A1, **TLN** crystals were soaked for 5 min in a cocktail of equal concentrations of the three ligands **1**–**3** (**Table** **1**). Three individual soaking sets B–D only contain a single ligand were set as references to demonstrate the ligand densities (Table 1). Data from 115 crystals were rapidly collected by automated MicroED overnight (Figure 1B) and after trimming (see “Section 4” for details) 65 structures were solved by molecular replacement without merging.^[^ ^34^ ^]^ The shape of F_o_–F_c_ maps in the binding site was overall well‐defined for all structures (literature^[^ ^26^ ^]^ and reference set B, Figure 2A), but only fit ligand **1** indicating that its binding affinity is much greater than those of ligands **2** and **3** (Figure S3, Supporting Information).

**Table 1:** Soaking setups for thermolysin and proteinase K.

Set/Grid	Protein	Ligand	Ligand concentrationa) [mM]	Soaking time	Soaking type
A1	**TLN**	**1** + **2** + **3**	5 + 5 + 5	5 min	Cocktail
A2b)		**1** + **2** + **3**	0.45 + 4.47 + 8.1	5 min	
B		**1**	5	2 h	Individual
C		**2**	5	2 h	
D		**3**	5	2 h	
E	PK	**4** + **5** + **6**	5 + 6.4 + 5	12 h	Cocktail
F		**4**	5	12 h	Individual
G		**5**	5	12 h	
H		**6**	5	5 min	

a) The final ligand concentration in soaking.

b) Set A2 was repeated twice to collect more datasets. Thermolysin was abbreviated as “**TLN**”, proteinase K was abbreviated as “**PK**”.

In a second cocktail (set A2), ligands **1**–**3** were mixed in ≈1:10:20 ratios (Table 1). The concentrations were set referring to the binding affinities calculated by AutoDock Vina,^[^ ^35^, ^36^ ^]^ i.e., −12.3 kcal mol^−1^ for **TLN**‐**1**, −6.1 kcal mol^−1^ for **TLN**‐**2**, and −4.3 kcal mol^−1^ for **TLN**‐**3**. A total of 102 structures were solved (Figure S4, Supporting Information) from 225 datasets, of which, 53 structures were found to exhibit F_o_–F_c_ density in their binding sites (Figure 2 and 4A). The data quality in set A2 was lower than in set A1, likely due to the presence of crystals binding different ligands (reference sets B‐D, Figure 2). Despite these limitations, the maps retained distinct ligand shapes (Figure 2). For example, in maps from crystals where ligand **1** was predominantly bound, the tryptophan is clearly visible but the map for the l‐ramnopyranoside moiety is weak (Figure 2A), possibly due to an increased flexibility or competition from ligands **2** and **3** (Figure S6A, Supporting Information). The density of ligand **2** in set A2 showed better occupancy than previously observed^[^ ^27^ ^]^ (reference set C, Figure 2B). The density of ligand **3** in sets A2 and D was generally complete and showed high consistency (Figure 2C). The terminal amine was visible in set D but missing in set A2, likely due to their resolution differences (Figure S6C, Supporting Information). Ligand **2** showed moderate binding affinity, as more **TLN**‐**2** complexes were found in set A2 despite using half the soaking concentration of ligand **3**. Although ligand 3 occupies the orthosteric site, **TLN**‐3 shows no conserved binding geometry, suggesting its association is likely driven by nonspecific diffusion rather than by directional contacts with the protein.^[^ ^28^ ^]^ MST measurements of *K*_D_ values for ligands **1**–**3** were 345 nM for ligand **1**, 4 μM for ligand **2**, and N.A. for ligand **3**, showing consistency with literature^[^ ^29^, ^30^ ^]^ and validation of MicroED results (Figure 4A,B).

### 2.2 Unknown Protein–Ligand Binding: Proteinase K in Complex with Ligands **4**–**6**

Proteinase K (**PK**) is a serine proteinase originally purified from *Tritirachium album*,^[^ ^37^ ^]^ featuring an active site with oxyanion triad composed of Asp144, His174, and Ser329 residues (Figure S2B, Supporting Information).^[^ ^33^ ^]^ **PK** is known to be inhibited by many ligands but lack published structures in literature, for example, ligand **4** can form an O—S bond with Ser329 residue using the same mechanism as phenylmethylsulfonyl fluoride (PMSF)^[^ ^38^, ^39^, ^40^ ^]^; ligand **5** diisopropyl can phosphorylate the Ser329 residue via an O—P bond;^[^ ^41^ ^]^ ligand **6** is a substrate‐mimic haloketone specifically designed for **PK**.^[^ ^42^ ^]^ Upon releasing the halogen atom (Cl), it forms a C—N bond between methylene and imidazole group in His174 residue, and a C—O bond between ketone carbonyl carbon and Ser329 residue. The complex structure between **PK** and ligand **6** has been deposited in PDB database (PDB entry: 4ZAR) but not published, the density map contains notably negative values near His174 residue promoting further improvements.

We aim to apply the above workflow (Figure 1) to elucidate protein–ligand binding in PK. When **PK** is cocktail soaked with ligands **4**–**6**, the resulting **PK**‐**4**, **PK**‐**5**, and **PK**‐**6** complexes irreversibly formed and coexisted in the mixture (Figure S2B, Supporting Information). Ratio of each component is determined by binding affinities (*K*_D_) and ligand concentrations. In set E, **PK** crystals were soaked for 12 h in a cocktail of ligands **4**–**6** in equal concentrations (Table 1). The extended incubation time was determined from pilot experiments to ensure a minimal disruption of the crystal lattice and a maximal ligand occupancy in **PK**. Three individual soaking sets F–H were set as references (Table 1). A total of 103 datasets were collected using automated MicroED (Figure 1B). The 82 datasets retained for further analysis (Figure S5, Supporting Information; see “Section 4” for details) yielded 62 structures by molecular replacement without merging.^[^ ^43^ ^]^ The three ligands were unambiguously identified in the F_o_–F_c_ maps (Figure 3 and 4C).

We compared the F_o_–F_c_ maps in the binding site in cocktail set E with those from the crystals where a single ligand was individually soaked (reference sets F–H Figure 3). Overall, the maps are clearer when soaking with a single ligand compared to the cocktail soaks, albeit at a similarly weaker counter level (Figure 3A). For example, the phenyl group of ligand **4** is partially missing in the cocktail soak, but its distinctive shape and orientation still allow unambiguous identification. The ligand in the cocktail‐soaked structure refines to ≈0.5 Å all‐atom r.m.s.d of its arrangement in the individually soaked sets; Figure S7A, Supporting Information). The density of ligand **5** was complete and consistent in both sets E and G (Figure 3B), and the structures refined from these datasets showed identical binding geometries (Figure S7B, Supporting Information). In set E, the density of ligand **6** was complete and superior to the unpublished PDB structure 4ZAR (RMSD: 0.23 Å). Two covalent bonds between Ser329 and His174 residues were clearly resolved from F_o_–F_c_ maps (Figure 3C), exactly consistent with the initial design of this inhibitor.^[^ ^42^ ^]^ In set H, the density of ligand **6** was also complete, but the His174 exhibited ≈2.9 Å shifts compared to set E; the density for Cl atom was visible. A noncovalent binding pose was occasionally observed in this case, where only noncovalent interactions were found between His174 and methylene (4.3 Å) and Ser329 and ketone carbonyl (3.0 Å). This alternative binding geometry might be caused by pH differences, which slowed the reaction rate of ligand **6** on PK.

Quantifying of each complex type in the mixture allowed for a quantitative comparison of their binding affinities: ligand **6** binds stronger than ligand **5**, which in turn exhibits greater affinity than ligand **4** (Figure 4C). Since the three ligands were mixed in approximately equal concentrations, the most frequently observed ligand **6** exhibited the highest binding affinity among the three. Ligand **5** showed slightly higher affinity than ligand **4**, but their counts are close, indicating comparable *K*_D_ values. MST measurements of K_D_ constants for ligands **4**–**6** were 260 μM for ligand **4**, N.A. for ligand **5**, and 18 μM for ligand **6**. Ligand **5** showed high activity in an aqueous environment and was degraded before a successful MST measurement; however, the measured *K*_D_ values for ligands **4** and **6** were consistent with MicroED results (Figure 4C,D).

## 3 Discussion

Cocktail soaking combined with high‐throughput data collection can significantly accelerate drug discovery by allowing rapid crystallographic screening of ligands against target proteins.^[^ ^1^, ^2^, ^3^, ^4^, ^5^, ^6^, ^9^, ^10^ ^]^ In conventional SC‐XRD, which requires crystals at least ≈5 μm in size,^[^ ^14^ ^]^ the expected success rate of ligand binding is relatively low (≈1%) making it unsuitable for protein microcrystals smaller than this size threshold and also leads to significant waste of ligands. Weak‐binding ligands might remain undetected if they failed in competition with stronger ligands in the cocktail, or untested if limited datasets were collected.

In this study, we proposed a workflow integrating cocktail soaking and automated MicroED for crystallographic ligand screening and structural analysis of protein–ligand complexes directly from microcrystals (Figure 1). After cocktail soaking, the mixture contains multiple protein–ligand complexes, all the ligands can be determined after sampling a sufficient number of crystals. Their F_o_–F_c_ maps were complete and comparable to those in literature^[^ ^26^, ^27^, ^28^ ^]^ and reference sets (Figure 2 and 3). The ligand with the highest affinity is expected to form the most crystals after cocktail soaking. Quantifying the proportion of each complex allows the estimation of their relative binding affinities, which is consistent with literature^[^ ^29^, ^30^ ^]^ and MST measured of *K*_D_ constants (Figure 4).

Detailed structural analysis of thermolysin and proteinase K in complex with their respective ligands reveals their binding differences in the active sites. For **TLN**, the phosphoryl group of ligand **1** coordinated with Zn ion at ≈2 Å, anchoring it within the binding cleft. The rest of the molecule is involved in at least seven hydrogen bonds, two salt bridges, and one water bridge. Similarly, the sulfanyl group of ligand **2** is coordinated with the Zn ion at ≈2.3 Å, forming 4 hydrogen bonds, 1 salt bridge, and 1 T‐shaped *pi*‐stacking. There is no conserved binding pose observed for ligand **3**, and its orientation appeared flexible within the binding pocket, which can be temporarily stabilized by a hydrogen bond with Asn344 or Arg435 or a water molecule. As for **PK**, ligand **4** formed an O—S covalent bond with the Ser329 residue. Its sulfonyl group formed at least 3 hydrogen bonds within the pocket. The rest of molecule (*e.g.* 2‐aminoethyl benzyl group) might form a weak hydrogen bond with Gly239 residue (≈3.6–3.8 Å) with minimum structural constraints. This increased rotational freedom likely explained the incomplete densities observed in F_o_–F_c_ maps (Figure 3A). Ligand **5** formed an O—P covalent bond with Ser329 residue, the phosphoryl group involved in four hydrogen bonds anchoring it within the binding pocket. As mentioned, ligand **6** displayed dual binding poses in sets E and H. In set E, ligand **6** formed 2 covalent bonds between His174 and methylene, Ser329 and ketone carbonyl carbon, which mimics the transition state of **PK** bound with substrate. This binding blocks the entry tunnel via at least eight hydrogen bonds and one water bridge. In set H, ligand **6** was noncovalently interacted with at least ten hydrogen bonds similar to set E.

The primary aim of this study is to develop a rapid crystallographic ligand screening approach, allowing the application of cocktail soaking into microcrystals. Because automated MicroED requires only 7 min per crystal, faster than a typical SC‐XRD experiment conducted at synchrotron,^[^ ^44^ ^]^ more datasets can be collected within the same time. High‐throughput data collection allows highly stringent data screening to focus on high‐quality datasets from crystals where ligands may be bound with high occupancy. Those datasets were subsequently trimmed by strict criteria (See “Section 4” for details) prioritizing crystals with better occupancy over high resolution. Most of the trimmed datasets enabled for unambiguous structural identification and refinement, however, cases where the same crystal binds several different ligands may be addressed using PanDDA in an “event map” approach,^[^ ^45^ ^]^ or as demonstrated by substituting with a better crystal with higher occupancy from the high‐throughput datasets. Weakly diffracting crystals were excluded to leave only high‐quality data solved for unambiguous structural determination (Figure 2 and 3).

This workflow offers future opportunities for drug discovery and development, such as the identification of novel drugs with superior binding compared to the biogenic ligands or existing drugs. It offers an alternative approach for evaluating ligand binding affinities, particularly for unstable compound like ligand **5**, for which directly measuring its *K*_D_ values is challenging (Figure 4C,D). Additionally, this method can yield a structure from each crystal, enabling characterization of the dynamics of protein–ligand interactions when many datasets are available (Figure 3C). In the follow‐up experiments, more ligands can be introduced at the cost of requiring longer data collection time. Due to resolution limits, ligands selected for cocktail soaking must exhibit diverse shapes following the established literature practices.^[^ ^1^, ^2^, ^3^, ^4^, ^5^, ^6^, ^9^, ^10^ ^]^ More structurally similar ligands can be tested in the future, given the resolution enhancement using crystal fragmentation^[^ ^34^ ^]^ or focused‐ion‐beam milling.^[^ ^46^, ^47^ ^]^ Finally, multiple mutated protein crystals can be combined and soaked with ligands to investigate essential molecular contacts.

## 4 Experimental Section

4.1

4.1.1

##### Materials

Thermolysin (*Bacillus thermoproteolyticus rokko*) was purchased from MedChemExpress. Proteinase K (*Tritirachium album*) was purchased from Sigma Aldrich. Ligands **1**, **3**, and **6** were purchased from Sigma Aldrich; ligand **2** was purchased from MedChemExpress. Ligand **4** was purchased from Focus Biomolecules. Ligand **5** was purchased from Thermo Scientific Chemicals.

##### Protein Crystallization

Crystallization of **TLN** and **PK** followed previously reported procedures.^[^ ^34^, ^43^ ^]^ **TLN** was dissolved at a concentration of 80 mg mL^−1^ in 45% dimethyl sulfoxide, 50 mM Tris–HCl (pH 7.5), and 2.5 m cesium chloride. Sitting drops were set up by mixing 0.5 μL protein solution and 1.5 μL water and equilibrating over 300 μL water at room temperature. **PK** was dissolved at a concentration of 25 mg mL^−1^ in 50 mM Tris–HCl (pH 8.0) and mixed with an equal volume of 1.25 m ammonium sulfate (pH 6.5) at room temperature. Microcrystals formed overnight, see Figure S8, Supporting Information.

##### Grid Preparation

Crystal slurries of **TLN** and **PK** were diluted and fragmented by sonicating for 20 s in an FS60 ultrasonic cleaner (Fisher Scientific) with a cold‐water bath.^[^ ^34^ ^]^ The resulting crystal slurries were soaked with ligands in different sets (Table 1).

Holey carbon grids (Quantifoil, R2/2 Cu200) were glow‐discharged for 30 s at 15 mA in negative mode. Blotting and vitrification were conducted using a Leica GP2 plunge freezer set at 20 °C and 90% humidity (Figure 1A). For each grid, 3 μL of crystal slurry was deposited on the carbon side and was gently blotted for 30 s from the copper side. Grids A1‐H (corresponding to sets A1‐H, Table 1) were plunged into liquid ethane and transferred to liquid nitrogen for storage.

##### Automatic MicroED Data Collection

MicroED data were collected with SerialEM on a Falcon 4i camera (4096 × 4096 pixels) mounted in a 300 keV Titan Krios Cryo‐TEM (Thermo Fisher) equipped with a Selectris energy filter.^[^ ^24^, ^25^, ^46^, ^47^ ^]^ Screening and data collection were accomplished using *in‐house* SerialEM scripts: first, the atlas of whole grid was montaged under the imaging mode (LM 155×). Around 100 grid squares with proper blotting were selected and montaged at higher magnification (SA 2250×) taking ≈7.5 h. Crystals with light contrast to the carbon film were typically thin and were chosen from montaged maps (≈1000 crystals).

The diffraction mode is configured with a gun lens 8, spot size 11, 50 μm C2 aperture, 20 eV energy filter slit, and 1.8 or 2.5 m camera lengths.^[^ ^46^ ^]^ Around10 μm sized parallel electron beam and a 100 μm SA aperture were used to result in a flux density of ≈0.002 e^−1^ (Å^2^·s)^−1^ on samples.^[^ ^46^ ^]^ All the selected crystals were tested with 1 s exposure time at 0° tilt in the diffraction mode to assess resolution. Only the best 100 crystals with resolutions better than 3.5 Å (Figure 1B) were retained for data collection. Eucentric heights were automatically calibrated to maintain the crystals inside the SA aperture during the continuous rotation. A typical data collection used a constant rotation rate of ≈0.2° s^−1^ over an angular wedge of 100° from −50° to +50° in 420 s exposure time. Overnight automatic data collection was applied for grids A1, A2, and E (Table 1), which typically yielded ≈100 datasets per grid. Manual data collection was performed for grids B–D and F–H (Table 1) using the same settings, which typically resulted in 10–15 datasets per grid.

##### MicroED Data Processing

Electron‐counted MicroED data were saved in electron event representation (EER) format and converted to super marty view (SMV) format using mrc2smv (https://cryoem.ucla.edu/downloads).^[^ ^47^, ^48^ ^]^ The converted frames were indexed and integrated in X‐ray detector software (XDS),^[^ ^49^, ^50^ ^]^ with the resolution cutoff chosen where I/σ ≥1. Datasets with resolution <3.5 Å, completeness <75% were discarded. The remaining datasets were scaled in XSCALE^[^ ^50^ ^]^ and converted to mcLaughlin, terry, zelinka (MTZ) format using XDSCONV.^[^ ^50^ ^]^ The structures were solved by molecular replacement using Phaser^[^ ^51^ ^]^ with electron scattering factors and unliganded PDB structures 5K7T and 6PKR as search models for **TLN** and **PK**, respectively,^[^ ^34^, ^43^ ^]^ and refined using phenix.refine (Figure 2 and 3).^[^ ^52^, ^53^ ^]^ Ligands were identified based on distinctive density shapes from initial F_o_–F_c_ maps calculated without ligands, comparing to those from literature^[^ ^26^, ^27^, ^28^ ^]^ and individual soaking references (Figure 2 and 3). Ligands were finally added and refined to yield PDB structures for **TLN**‐**1** to **TLN**‐**3** and **PK**‐**4** to **PK**‐**6** (Table S1–S6, Supporting Information).

##### MST

Labeled **TLN** was prepared in 25 mM HEPES (pH 7.4), 150 mM NaCl, labeled with RED‐NHS dye (NanoTemper), and purified to a concentration of 5 mM. Ligands **1**–**3** were prepared in 25 mM HEPES (pH 7.4), 150 mM NaCl at a concentration of 2–5 mM, then diluted to a series of concentrations from mM to μM. The diluted ligands were mixed with labeled protein in 1:1 ratio, which were loaded to monolith capillaries (NanoTemper). MST was measured at 37 °C for 10 s with two repeats. MST measurements for **PK** and ligands **4**–**6** followed the same procedure. DMSO was used in preparing 25 μM ligand **6** due to its low water solubility.

The normalized fluorescence signal (*F*_Norm_) versus the concentration of ligands [*L*] measured with MST are plotted in Figure 4B,D. The binding affinity (*K*_D_) was calculated by fitting *F*_Norm_ and [*L*] to the Equation (1) below

(1) $$F_{\text{Norm}}=F_{\text{Unbound}}+\left(F_{\text{Bound}}-F_{\text{Unbound}}\right)\times \frac{\left[L\right]+\left[P\right]+K_{D}-\sqrt{{\left(\left[L\right]+\left[P\right]+K_{D}\right)}^{2}-4\left[L\right]\left[P\right]}}{2\left[P\right]}$$

where $F_{\text{Norm}}$ is the normalized fluorescence signal, $F_{\text{Unbound}}$, *F*_Norm_ is the signal of the target protein alone; $F_{\text{Bound}}$: *F*_Norm_ signal of the complex. The final concentrations of the ligand and the target protein are denoted [L] and [P], respectively, and $K_{D}$ is the binding affinity or dissociation constant.

## Conflict of Interest

The authors declare no conflict of interest.

## Author Contributions

**Jieye Lin**: data curation (lead); formal analysis (lead); software (lead); validation (lead); visualization (lead); writing—original draft (lead). **Marc J. Gallenito**: data curation (supporting); validation (supporting); writing—review & editing (supporting). **Johan Hattne**: supervision (supporting); validation (supporting); writing—review & editing (supporting). **Tamir Gonen**: conceptualization (lead); funding acquisition (lead); project administration (lead); supervision (lead); writing—review & editing (lead).

## Supporting information

Supplementary Material

## Data Availability

The data that support the findings of this study are available in the supplementary material of this article.
